# *CircATRNL1* promotes epithelial–mesenchymal transition in endometriosis by upregulating Yes-associated protein 1 in vitro

**DOI:** 10.1038/s41419-020-02784-4

**Published:** 2020-07-29

**Authors:** Dandan Wang, Yajuan Luo, Guangwei Wang, Qing Yang

**Affiliations:** Department of Obstetrics & Gynecology, Shengjing Hospital of China Medical University, No.36 Sanhao Street, Heping District, Shenyang, 110004 PR China

**Keywords:** Epithelial-mesenchymal transition, Mechanisms of disease, miRNAs

## Abstract

Endometriosis is a common and benign gynecological disorder but exhibits malignant features. However, the underlying pathogenesis and pathophysiology of endometriosis remain unclear. Circular RNAs have been demonstrated to participate in the occurrence and progression of multiple diseases. This study was aimed to explore the roles of *circATRNL1* in endometriosis in vitro. Based on the results of reverse transcription-quantitative polymerase chain reaction analysis, we found significant upregulation of *circATRNL1* and Yes-associated protein 1 (*YAP1)*, while downregulation of *miR-141-3p* and *miR-200a-3p* in ectopic tissues compared to eutopic tissues. The immunohistochemistry and western blot analysis showed differentially expressed epithelial–mesenchymal transition (EMT) markers between EuEM and EcEM tissues. The in vitro assays indicated that overexpression of *circATRNL1* could promote the proliferation, migration, and invasion of Ishikawa cells, and induce EMT process, while *circATRNL1* silencing showed the opposite effect. The mechanical investigation indicated that *circATRNL1* upregulated *YAP1* by sponging *miR-141-3p* and *miR-200a-3p*. Gain-of-function assays validated the inhibitory function of *miR-141-3p* and *miR-200a-3p* in endometriosis. The results of rescue assays confirmed the function of *circATRNL1*–*miR-141-3p*/*miR-200a-3p*–YAP1 axis on Ishikawa cells. Our findings demonstrate that abnormal upregulation of *circATRNL1* regulates cell proliferation and motility and promotes EMT process via the *miR-141-3p/miR-200a-3p*–*YAP1* axis in vitro, which could contribute to the progression of endometriosis.

## Introduction

Endometriosis is a common and refractory estrogen-dependent gynecological disorder associated with dysmenorrhea, pelvic pain, and infertility^1–4^. Although benign, this chronic and multifactorial disease exhibits tumor-like biological behaviors and affects the physical and mental health of affected women^5^. However, the underlying pathogenesis and pathophysiology of endometriosis remain unclear.

The epithelial–mesenchymal transition (EMT) is a special biological process in which immotile epithelial cells convert into highly motile mesenchymal cells with migratory and invasive properties during embryonic development, chronic inflammation, tissue construction, fibrosis formation, and cancer metastasis^6–9^. The EMT is characterized by decreased expression of epithelial markers, such as E-cadherin, and increased expression of mesenchymal markers, such as N-cadherin and vimentin^10^. Many studies have documented the involvement of the EMT in the pathogenesis and development of endometriosis^7–9,11^. Chen et al.^12^ first found decreased E-cadherin and increased vimentin expression in ectopic glandular epithelial cells in adenomyosis. Another study showed that E-cadherin-negative cells were frequently observed in endometriotic tissues, whereas N-cadherin, Twist, Slug, and Snail were all upregulated in endometriosis compared with that in the healthy endometrium^7^. The mesenchymal marker zinc finger E-box-binding homeobox 1 (ZEB1), a transcriptional repressor of E-cadherin, is also upregulated in endometriotic lesions^13^. Several signal pathways, including the Wnt/β-catenin pathway^11^, the Notch pathway^14^, and the transforming growth factor (TGF)-β/Smad pathway^15^, have been reported to regulate the EMT in endometriosis. Moreover, other factors, including estrogen^11,12^ and hypoxia-inducible factor-1α^16^, may act as powerful inducers of the EMT in endometriosis. Changes in EMT marker proteins have been detected in ectopic epithelial cells, resulting in enhanced migration and invasion as a prerequisite for the establishment of endometriotic lesions.

Recent studies have highlighted the regulatory mechanisms through which noncoding RNAs (ncRNAs) participate in the occurrence and progression of multiple diseases^17–19^. Circular RNAs (circRNAs), as a distinct type of ncRNAs, have a covalently closed continuous loop and display resistance to exonuclease digestion^20,21^. CircRNAs show highly stable expression in eukaryotic cells, are predominantly localized in the cytoplasm, and are evolutionarily conserved across species, suggestive of their important regulatory roles^17^. As competing endogenous RNAs (ceRNAs), circRNAs may sequester microRNAs (miRNAs) with their own miRNA response elements and function as miRNA “sponges” to strongly suppress miRNA activity, thereby increasing the levels of miRNA target genes and mediating cellular biological behaviors involved in human diseases^17,22^. For example, *ciRS-7* contains more than 70 selectively conserved miRNA target sites and regulates the initiation and progression of various malignancies in an *miR-7*-dependent manner^23,24^. In addition, *circ-PVT1* facilitates paclitaxel resistance by upregulating ZEB1 via *miR-124-3p* in gastric cancer^25^. Overexpression of *circ-0001649* inhibits the proliferation and migration of hepatocellular carcinoma (HCC) in vitro and in vivo by serving as a ceRNA to sponge *miR-127-5p*, *miR-612*, and *miR-4688*^26^. These findings suggest that circRNAs may be potential biomarkers and therapeutic targets in the diagnosis and treatment of multiple diseases. However, our understanding of the relationships between circRNAs and endometriosis remains limited, and translating current circRNA-related research into clinical practice is challenging.

Our previous study showed that circRNAs are aberrantly expressed in the ectopic endometrium (EcEM) compared with that in paired eutopic endometrium (EuEM) samples and demonstrated that *hsa-circ-0020093*, derived from the *ATRNL1* gene locus (thus, designated *circATRNL1*), was upregulated in ovarian endometriosis^27^. Accordingly, in this study, we aimed to investigate the roles of *circATRNL1* in endometriosis in vitro. Our findings provided important insights into the roles of *circATRNL1* in the EMT in endometriosis and may aid in developing suitable therapeutic strategies.

## Materials and methods

### Ethics

This study was approved by the Ethics Committees of Shengjing Hospital of China Medical University (ethics no. 2018PS504K), and written informed consent was obtained from all patients before surgical procedures and sample collection.

### Clinical specimens and cell culture

Snap-frozen cyst walls of ovarian endometriomas and matched EuEM samples from the same patient were collected from 60 women (20–44 years old) with a laparoscopic and histological diagnosis of stage III/IV endometriosis according to the Revised American Society for Reproductive Medicine classification system (rASRM; 1997). All patients had regular menstrual cycles (21–35 days), and none had received any hormone therapy for at least 6 months prior to the operation. All EuEM samples were collected during the proliferative phase of the menstrual cycle, as confirmed by both the date of the last menstrual period and histological diagnosis.

Ishikawa cells (a well-differentiated endometrial adenocarcinoma cell line) and HEK-293T cells were purchased from Huiying Biological Technology Co. Ltd (Shanghai, China) and cultured in modified Eagle’s medium (Gibco, USA) with 10% fetal bovine serum (FBS; Bioind, Israel), 100 μg/mL streptomycin, and 100 IU/mL penicillin (Beyotime, Shanghai, China) in a humidified atmosphere with 5% CO_2_ at 37 °C.

### Immunohistochemistry (IHC)

Immunohistochemical staining was performed on paraffin-embedded tissues. Three-micrometer-thick sections were cut and placed on glass slides. Tissues were deparaffinized in xylene and rehydrated in an ethanol gradient. Antigen retrieve was performed in 10 mM Sodium Citrate Buffer (pH 6.0). Overall, 3% H_2_O_2_ was used to block endogenous peroxidase for 15 min. Nonspecific background staining was then blocked by incubation with goat serum (Solarbio, China) for 15 min. The slices were incubated with primary antibodies overnight at 4 °C and horseradish peroxidase (HRP)-conjugated secondary antibodies at 37 °C for 1 h. All primary and secondary antibodies were as follows: rabbit polyclonal anti-E-cadherin (P12830) (Cat. No.WL01482, 1:200; Wanleibio, China), mouse monoclonal anti-N-cadherin (P19022) (1:300; Proteintech, Wuhan, China), rabbit polyclonal anti-vimentin (P08670) (1:200; Proteintech), mouse monoclonal anti-YAP1 (P46937) (1:200; Proteintech), rabbit polyclonal anti-ZEB1 (P37275) (1:200; Proteintech), goat anti-mouse IgG H&L antibody (Product # 31321, 1:500; Thermo Fisher, USA), and goat anti-rabbit IgG H&L antibody (Product # 31460, 1:500; Thermo Fisher, USA). All slides were incubated with DAB (Solarbio, China) and then counterstained with hematoxylin (Solarbio, China) before they were dehydrated and mounted. Finally, the staining was visualized under a light microscopy (Olympus, Japan). Three randomly selected microscopic fields (×400 magnification) were photographed and the mean optic density in each field was counted and analyzed using Image-Pro Plus software (Media Cybernetics, USA).

### Plasmid construction and cell transfection

Three interference sequences targeting *circATRNL1* were constructed and inserted into the *EcoR*I–*BamH*I site of the pHBLV-U6-MCS-CMV-ZsGreen-PGK-Puro vector. The sequences were as follows: #1, 5′-ATAGTTGGCAAGGTTAACAGAACCT-3′; #2, 5′-TTGGCAAGGTTAACAGAACCTTCTG-3'; #3, 5'-CAATGATAGTTGGCAAGG TTAACAG-3′; For lentivirus-mediated overexpression of *circATRNL1* in Ishikawa cells, full-length *circATRNL1* was inserted into the pHBLV-CMV-circ-MCS-EF1-zsgreen-T2A-puro vector. Lentivirus packaging, cell infection, and selection of puromycin-resistant cells were performed according to the instruction manual provided by Hanbio Biotechnology (Shanghai, China). The sequences of miRNA mimics, miRNA inhibitors, and siRNAs were as follows: *miR-141-3p* mimic, forward 5′-UAACACUGUCUGGUAAAGAUGG-3′ and reverse 5′-AUCUUUACCAGACAGUGUUAUU-3′; *miR-141-3p* inhibitor, 5′-CCAUCUUUACCAGACAGUGUUA-3′; *miR-200a-3p* mimic, forward 5′-UAACACUGUCUGGUAAACG AUGU-3′ and reverse 5′-AUCGUUACCAGACAGUGUUAUU-3′; *miR-200a-3p* inhibitor, 5′-AC AUCGUUACCAGACAGUGUUA-3′; Yes-associated protein 1 (*YAP1*) siRNA (#1), forward 5′-CUGCCACCAA GCUAGAUAATT-3′ and reverse 5′-UUAUCUAGCUUGGUGGCAGTT-3′; (#2), forward 5′-GGUGAUACUAUCAACCAAATT-3′ and reverse 5′-UUUGGUUGAUAGUAUCACCTT-3′; (#3), forward 5′-GACGACCAAUAGCUCAGAUTT-3′ and reverse 5′-AUCUGAGCUAUUGGUCGUCTT-3′; negative control (NC), forward 5′-UUCUCCGAACGUGUCACGUTT-3′ and reverse 5′-ACGUGACACGUUCGGAG AATT-3′; inhibitor negative control (si-NC), 5′-CAGUACUUUUGUGUAGUACAA-3′. All of these RNA oligos were purchased from GenePharma (Suzhou, China) and were transfected into Ishikawa cells using Lipofectamine 3000 Reagent (Invitrogen, USA).

### RNA isolation and reverse transcription-quantitative polymerase chain reaction (RT-qPCR)

Total RNA was isolated from human tissues and cultured cells with TRIzol reagent (TaKaRa, Japan). RNA quantity and quality were measured using a Nanophotometer N50 (Implen, Germany). Only when the ratio of the absorbance at 260 and 280 nm was between 1.8 and 2.2 was the total RNA sample considered acceptable. All RNA samples were stored at −80 °C until further use. The expression levels of circRNAs were evaluated by RT-qPCR using a One-step SYBR PrimeScript RT-PCR kit (cat. no. RR660A; TaKaRa) on an Applied Biosystems 7500 FAST instrument (Applied Biosystems, Foster City, CA, USA). The cycling program was initiated at 42 °C for 5 min and 95 °C for 10 s, followed by 40 cycles of 95 °C for 5 s and 60 °C for 34 s. An Mir-X miRNA First-strand Synthesis Kit (cat. no. 638315; TaKaRa) and PrimeScript RT Reagent Kit with gDNA Eraser (cat. no. RR047A; TaKaRa) were used for first-strand synthesis of miRNAs and mRNAs, respectively. Then, the amplification conditions were carried out using SYBR Premix Ex TaqII (cat. no. RR820A; TaKaRa) according to the manufacturer’s instructions, as follows: 95 °C for 30 s, followed by 40 cycles of 95 °C for 5 s and 60 °C for 34 s. All PCR primers were designed and synthesized by Sangon Biotech (Shanghai, China) and were as follows: *circATRNL1*, forward 5′-GCAATGATAGTTGCAAGGTTAAC-3′, reverse 5′-GCCTTCAATGAGC CAAGTACA-3′; *miR-141-3p*, forward 5′-GCGCTAACACTGTCTGGTAAAGATGG-3′; *miR-200a-3p*, forward 5′-GCGTAACACTGTCTGGTAACGATGT-3′; *YAP1*, forward 5′-CCGTTTCCCAGACTACCTT-3′, reverse 5′-TTGGCATCAGCTCCTCTC-3′. Each sample had three individual technical replicates. The threshold cycle method (2^−ΔΔCT^) was used to calculate relative quantification of expression levels compared with the internal standard.

### Cell proliferation assay

After transfection, Ishikawa cells were seeded on 96-well flat-bottomed microplates at a density of 5000 cells/well. The culture medium was regularly replaced. For analysis of cell proliferation, 100 μL of Cell Counting Kit-8 (CCK-8) reagent (Dojindo, Japan) was added into each well at different time points (0, 24, 48, 72, and 96 h), followed by incubation for 1 h at 37 °C. The absorbance of each well at 490 nm was observed and measured with a Universal Microplate Spectrophotometer (Bio-Tek Instruments, Inc., Winooski, VT, USA).

### Cell invasion and migration assays

Cell invasion assays were performed using a Transwell system (Corning, NY, USA). After dilution with serum-free medium, 100 μL Matrigel (cat. no. 356234; Corning) was added to each transwell chamber and coagulated for 4 h at 37 °C. After treatment for 48 h, Ishikawa cells were washed and cultured with serum-free medium for 12 h. Next, a 200-μL aliquot of the above single-cell suspension (2 × 10^4^ cells) was placed into each upper chamber of a transwell plate. The lower chamber was filled with 700 mL of medium containing 10% FBS. After 24 h of incubation, a cotton swab was used to remove cells remaining in the upper chamber. The cells that invaded into the membrane were fixed in 4% formaldehyde (Solarbio, Beijing) for 30 min, and then stained with 0.1% crystal violet solution (Solarbio). Eight randomly selected microscopic fields were photographed with an Eclipse Ti-s microscope (Nikon, Japan), and the number of cells in each field was counted using Image-Pro Plus software (Media Cybernetics, USA). Cell migration assays were performed according to the same procedure, except that the Matrigel was omitted.

### Fluorescence in situ hybridization (FISH)

Hybridization was performed overnight at 37 °C with specific *circATRNL1* probes, sections were washed, and DAPI staining was performed for 20 min in the dark. The sections were observed under a Leica TCS SP2 AOBS Confocal (upright) fluorescence microscope (×40 objective, ×10 ocular), and images were acquired. Nuclei stained with DAPI appeared blue under UV excitation and contained *circATRNL1*, which was labeled with Cy3 (red emission), and miRNAs (*miR-141-3p* and *miR-200a-3p*) which were labeled with FITC (green emission). The sequence of probes used for FISH were as follows: c*ircATRNL1*, 5′-TTCTGTTAACCTTGCCAACTA-3′; *miR-141-3p*, 5′-CCATCTTTACCAGACAGTGTTA-3′; *miR-200a-3p*, 5′-CATCGTTACCAGACAGTGTTA-3′. All the probes synthesis and FISH detection were performed by Geneseed (Guangzhou, China).

### Dual-luciferase activity assay

pmiR-GLO-YAP1 wild-type or mutated vectors, miRNA mimics or negative control (Genepharm), and wild-type *circATRNL1* or mutated *circATRNL1* vectors (Ribobio, Guangzhou, China) were transfected into 293T cells. Firefly and Renilla luciferase activities were measured consecutively 24 h after transfection using Dual-Luciferase Reporter Assays (cat. no. E1910; Promega, Madison, WI, USA) according to the manufacturer’s instructions.

### Western blot analysis

Tissues and cell lysates were prepared with RIPA buffer (Beyotime) containing 10% phenylmethylsulfonyl fluoride. Next, a BCA Kit (Beyotime) was used to quantify protein concentrations. Samples were separated by sodium dodecyl sulfate-polyacrylamide gel electrophoresis on 10% gels (Beyotime) and transferred onto polyvinylidene difluoride membranes (Millipore, USA). The membranes were blocked with 5% skim milk for 2 h and incubated with primary antibodies at room temperature for 1.5 h or overnight at 4 °C. All primary and secondary antibodies were purchased from Proteintech (Wuhan, China). The antibodies were as follows: mouse monoclonal anti-E-cadherin (Cat.No. 60335-1-Ig, 1:2500), mouse monoclonal anti-N-cadherin (Cat.No. 66219-1-Ig,1:2000), mouse monoclonal anti-vimentin (Cat.No. 60330-1-Ig, 1:6000), mouse monoclonal anti-YAP1 (Cat.No. 66900-1-Ig,1:1000), rabbit polyclonal anti-ZEB1 (Cat.No.21544-1-AP, 1:1000), mouse monoclonal anti-glyceraldehyde 3-phosphate dehydrogenase (GAPDH) (P04406) (Cat.No.60004-1-Ig, 1:20000), mouse monoclonal anti-β-tubulin (P04350) (Cat.No. 66240-1-Ig, 1:20000), goat anti-mouse IgG H&L (Cat.No. SA00001-1 HRP conjugated; 1:5000), and goat anti-rabbit IgG H&L antibodies (Cat.No. SA00001-2, HRP conjugated; 1:5000). The immunoblot signal was quantified with ImageJ software.

### Statistical analysis

All experiments were performed in triplicate. The data are expressed as means ± standard errors of the means and analyzed by GraphPad Prism 7 (La Jolla, CA, USA). Differences between two groups were evaluated with Student’s *t* tests. Comparisons among three or more groups were performed by one-way analysis of variance and Tukey’s post hoc test. Each experiment was conducted triplicate. Correlations among *circATRNL1*, *miR-141-3p/miR-200a-3p*, and *YAP1* were analyzed with Spearman rank correlation. *P* values of less than 0.05 were considered statistically significant.

## Results

### CircATRNL1 was upregulated in ovary endometriosis tissues

**Fig. 1:**
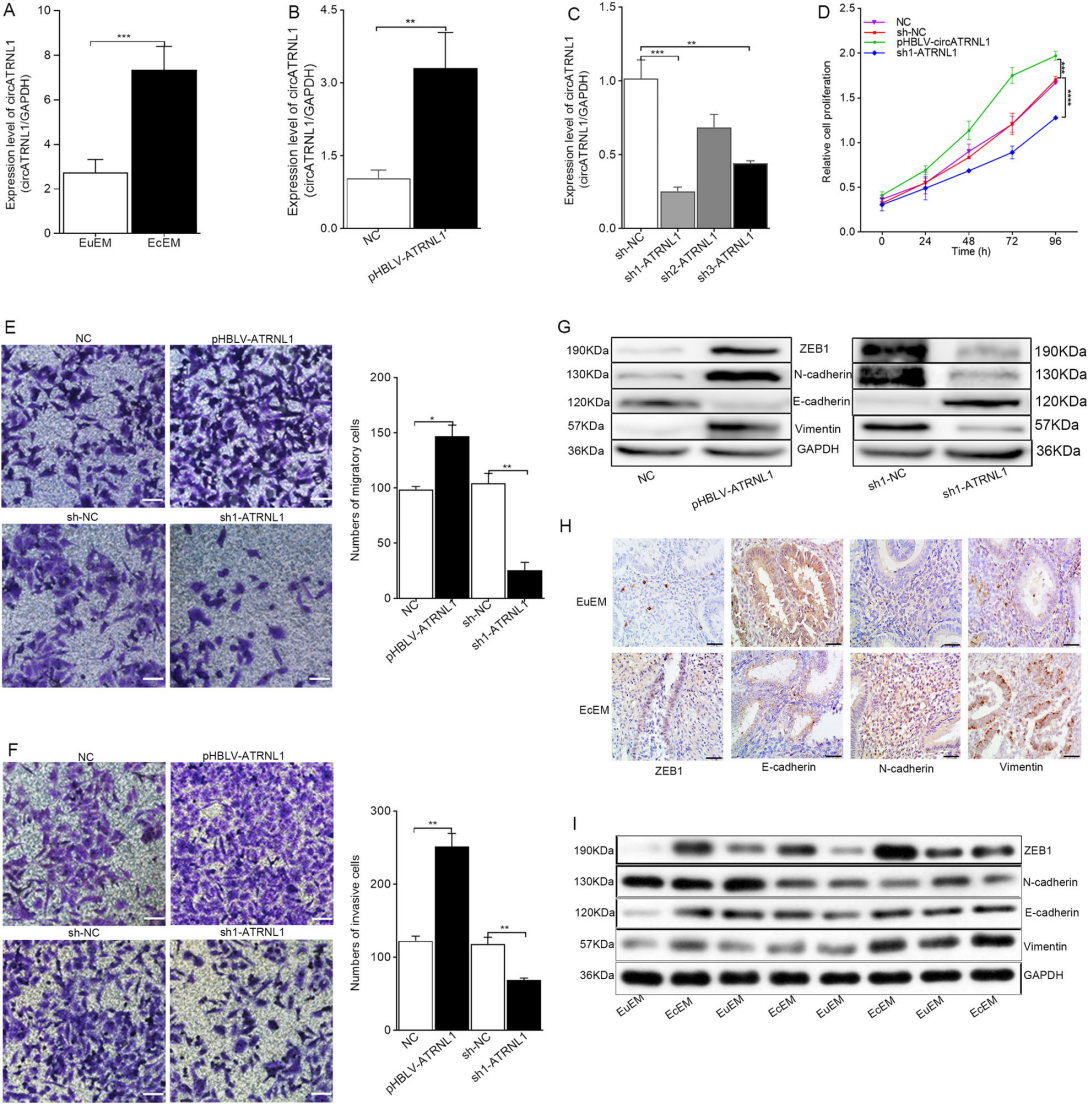
circATRNL1 regulates proliferation, migration, invasion, and EMT process in endometriosis. **a** The expression level of *circATRNL1* in 60 ovary endometriosis tissue samples was examined by using qRT-PCR. **b** *circATRNL1* was overexpressed in Ishikawa cells by transfecting with lentivirus-mediated vector pHBLV-ATRNL1. NC was used as a control. **c** *circATRNL1* was silenced in Ishikawa cells by transfecting with specific sh-ATRNL1. shRNA-NC was used as a control. **d** CCK-8 assays were conducted to determine the influence of *circATRNL1* on cell proliferation. **e** Transwell migration assays showed that *circATRNL1* regulated the migrative potential of Ishikawa cells. Photographs were taken at ×200 magnification. Scale bar represents 100 μm. **f** Transwell invasion assays showed that *circATRNL1* regulated the invasive potential of Ishikawa cells. **g** Western blot analysis was utilized to analyze the impacts of *circATRNL1* overexpression or knockdown on EMT progress in Ishikawa cells. **h** Immunohistochemistry of EMT markers in 60 EuEM and EcEM tissues. Original magnification: ×400. Scale bars represent 50 μm. **i** Western blot analysis of EMT markers in 60 EuEM and EcEM tissues. Assays were performed in triplicate. *P < 0.05, **P < 0.01, ***P < 0.001, ****P < 0.0001 represent statistical difference.

To validate the expression levels of *circATRNL1*, RT-qPCR was conducted in 60 EcEM and EuEM samples. The data showed that *circATRNL1* expression was significantly increased in ovary EcEM tissues compared with that in EuEM tissues (Fig. 1a, *P* = 0.0002). This suggested that *circATRNL1* may play a role in the development of endometriosis.

### Knockdown of circATRNL1 inhibited cell growth, migration, and invasion in vitro

To further explore the underlying functions of *circATRNL1* in endometriosis, we performed a series of validation assays by overexpressing and silencing *circATRNL1* in Ishikawa cells, respectively. The relative expression level of *circATRNL1* was validated by RT-qPCR. Importantly, the pHBLV-ATRNL1 vector upregulated *circATRNL1* in Ishikawa cells (Fig. 1b, *P* = 0.0071). Three specific lentivirus-mediated shRNAs (sh1-ATRNL1, sh2-ATRNL1, and sh3-ATRNL1) were transfected into Ishikawa cells to silence *circATRNL1* expression; sh1 showed the highest efficiency and was selected for further cellular experiments (Fig. 1c, *P* = 0.0009 for sh-NC vs. sh1, *P* = 0.0815 for sh-NC vs. sh2, *P* = 0.003 for sh-NC vs. sh3).

Subsequently, CCK-8 assays were conducted to determine the influence of *circATRNL1* on cell proliferation. Our results demonstrated that overexpression of *circATRNL1* significantly promoted the proliferation of Ishikawa cells (*P* = 0.0010), whereas *circATRNL1* knockdown decreased the growth of Ishikawa cells (Fig. 1d, *P* = 0.00005). We also observed that the migratory and invasive capabilities of Ishikawa cells were significantly enhanced by overexpression of *circATRNL2* (Fig. 1e, *P* = 0.0108 for NC vs. pHBLV-ATRNL1, *P* = 0.0025 for sh-NC vs. sh1-ATRNL1), but significantly inhibited by silencing of *circATRNL1* (Fig. 1f, *P* = 0.0025 for NC vs. pHBLV-ATRNL1, *P* = 0.0078 for sh-NC vs. sh1-ATRNL1). These results revealed that *circATRNL1* knockdown may efficiently reduce endometriosis by inhibiting cell growth, migration, and invasiveness.

### Knockdown of circATRNL1 inhibited EMT progression in endometriosis

To further detect whether *circATRNL1* promoted migration and invasion by facilitating the EMT in Ishikawa cells, we tested the expression of EMT hallmarks by western blot analysis. As shown in Fig. 1g, the levels of epithelial markers (E-cadherin) were enhanced, whereas the levels of mesenchymal markers (N-cadherin, vimentin, and ZEB1) were obviously decreased in *circATRNL1* knockdown Ishikawa cells. In contrast, the levels of EMT hallmarks showed the opposite changes in response to *circATRNL1* overexpression. Taken together, our findings demonstrated that *circATRNL1* expression was correlated with the EMT process in endometriosis. In addition, we tested the expression of EMT hallmarks by IHC and western blot analysis in 60 ovary EcEM tissues and EuEM tissues. The data showed that, compared with that in EuEM tissues, the levels of epithelial markers (E-cadherin) were decreased in EcEM tissues, whereas the levels of mesenchymal markers (N-cadherin, vimentin, and ZEB1) were obviously enhanced (Fig. 1h, i).

### miR-141-3p and miR-200a-3p were targets of circATRNL1

**Fig. 2:**
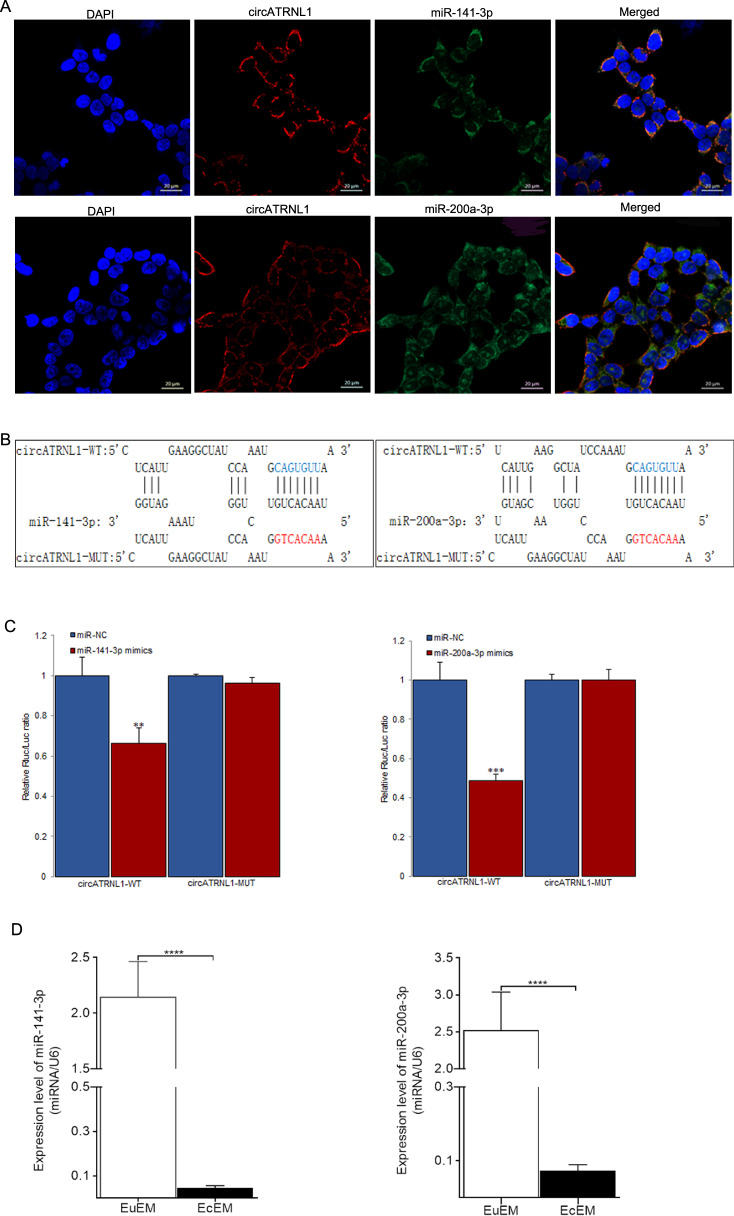
miR-141-3p and miR-200a-3p are targets of circATRNL1. **a** FISH assay was used to determine the location of *circATRNL1*, *miR-141-3p*, and *miR-200a-3p* in Ishikawa cells (blue, DAPI nuclear staining; red, *circATRNL1*; green, *miR-141-3p* and *miR-200a-3p*). Scale bars represent 20 μm. **b** The binding sites between wild-type *circATRNL1* or mutated *circATRNL1* and *miR-141-3p/miR-200a-3p* were predicted by using bioinformatics analysis. **c** Dual-luciferase reporter assays were carried out in HEK-293T cell to confirm the combination between *circATRNL1* and *miR-141-3p* mimics or *miR-200a-3p* mimics. **d** qRT-PCR analysis was carried to determine the expression pattern of *miR-141-3p* and *miR-200a-3p* in ovary endometriosis tissues. ****P < 0.0001 represents statistical difference.

Accumulating evidence has demonstrated that circRNAs act as ceRNAs to sponge miRNAs and modulate the expression of target genes at the post-transcriptional level. To confirm that *circATRNL1* exerted biological functions through acting as a ceRNA, we first performed FISH assays to identify the localization of *circATRNL1* in Ishikawa cells. *CircATRNL1* was mainly localized in the cytoplasm of Ishikawa cells (Fig. 2a). According to our previous bioinformatics prediction, *miR-141-3p* and *miR-200a-3p* may bind *circATRNL1* to affect the pathogenesis of endometriosis^27^. Furthermore, a luciferase reporter vector was designed and transfected into HEK-293T cells to facilitate the combination between *circATRNL1* and the two miRNAs (Fig. 2b). The results showed that both *miR-141-3p* mimics (*P* = 0.0078) and *miR-200a-3p* mimics (*P* = 0.0008) decreased the luciferase activity of wild-type *circATRNL1* (circATRNL1-WT), whereas the luciferase activity of mutated *circATRNL1* (circATRNL1-MUT) was not significantly altered (Fig. 2c). Subsequently, *miR-141-3p* (*P* < 0.00001) and *miR-200a-3p* (*P* < 0.00001) were shown to be downregulated in EcEM tissues compared with that in EuEM tissues (Fig. 2d). Accordingly, the expression correlation between circATRNL1 and *miR-141-3p* (*r* = −0.434, *P* = 0.001) and *miR-200a-3p* (*r* = −0.418*, P* = 0.001) was separately proved to be negative.

### miR-141-3p and miR-200a-3p inhibited EMT progression in endometriosis

**Fig. 3:**
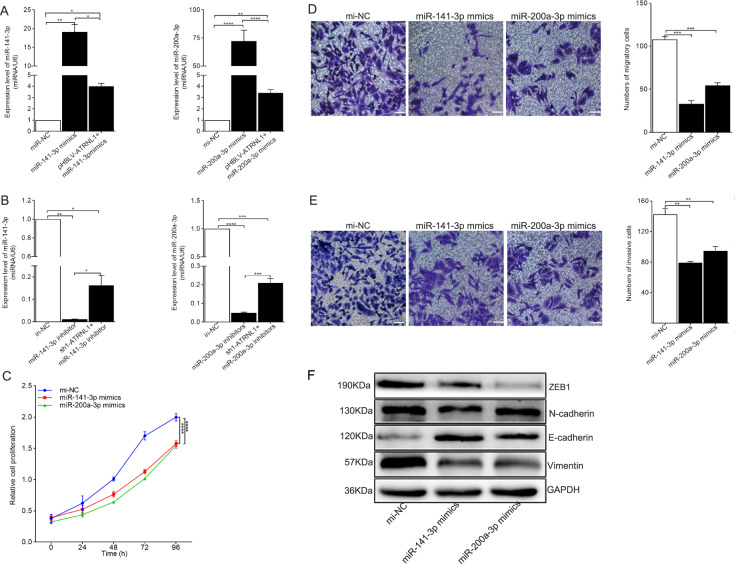
miR-141-3p and miR-200a-3p inhibited EMT process in endometriosis. **a** *miR-141-3p* and *miR-200a-3p* were overexpressed with *miR-141-3p* or *miR-200a-3p* mimics in Ishikawa cells, respectively, and be partly rescued by pHBLV-ATRNL1 co-transfection. mi-NC was taken as the control group. **b** *miR-141-3p* and *miR-200a-3p* were downregulated with *miR-141-3p* or *miR-200a-3p* inhibitors in Ishikawa cells, respectively, and be partly rescued by sh1-circATRNL1 co-transfection. in-NC was taken as the control group. **c**–**e** CCK-8 and transwell assays were separately carried out to identify the influence of *miR-141-3p* and *miR-200a-3p* mimics on cell proliferation, migration, and invasion. Photographs were taken at ×200 magnification. Scale bar represents 100 μm. **f** The effect of *miR-141-3p* and *miR-200a-3p* mimics on EMT progress in Ishikawa cells was evaluated through detecting the EMT marker proteins with western blot analysis. *P < 0.05, **P < 0.01, ***P < 0.001, ****P < 0.0001 represent statistical difference.

*miR-141-3p* and *miR-200a-3p* belong to the *miR-200* family, which modulates EMT progression in several diseases. Nevertheless, the specific roles of *miR-141-3p* and *miR-200a-3p* in endometriosis are still unknown. Accordingly, *miR-141-3p* mimics/inhibitors and *miR-200a-3p* mimics/inhibitors were transfected into Ishikawa cells for overexpression or knockdown of the corresponding target gene, with mi-NC and in-NC used as the control group. RT-qPCR was used to measure transfection efficiency after 48 h. Co-transfection with pHBLV-ATRNL1 or sh1-ATRNL1 rescued the efficiency of miRNA mimics (Fig. 3a, *P* = 0.0018 for *mi-NC* vs. *miR-141-3p* mimics, *P* = 0.0129 for *miR-141-3p* mimics vs. pHBLV-ATRNL1+ *miR-141-3p* mimics, *P* = 0.0111 for *mi-NC* vs. pHBLV-ATRNL1+ *miR-141-3p* mimics; *P* < 0.00001 for *mi-NC* vs. *miR-200a-3p* mimics, *P* < 0.00001 for *miR-200a-3p* mimics vs. pHBLV-ATRNL1+ *miR-200a-3p* mimics, *P* = 0.0025 for *mi-NC* vs. pHBLV-ATRNL1+ *miR-200a-3p* mimics) or inhibitors (Fig. 3b, *P* = 0.002 for *in-NC* vs. *miR-141-3p* inhibitors, *P* = 0.0105 for *miR-141-3p* inhibitors vs. sh1-ATRNL1+ *miR-141-3p* inhibitors, *P* = 0.0328 for *in-NC* vs. sh1-ATRNL1 + *miR-141-3p* inhibitors; *P* < 0.00001 for *in-NC* vs. *miR-200a-3p* inhibitors, *P* = 0.0002 for *miR-200a-3p* inhibitors vs. sh1-ATRNL1+ *miR-200a-3p* inhibitors, *P* = 0.0001 for *in-NC* vs. sh1-ATRNL1+ *miR-200a-3p* inhibitors). Similarly, CCK-8 assays and transwell migration and invasion assays were carried out to evaluate the influence of *miR-14-3p/miR-200a-3p* mimics on Ishikawa cells. As showed in Fig. 3c, cell proliferation was obviously inhibited by *miR-141-3p* mimics (*P* < 0.00001) and *miR-200a-3p* mimics (*P* < 0.00001). The results of transwell experiments demonstrated that the migratory (Fig. 3d, *P* = 0.0001 for mi-NC vs. *miR-141-3p*, *P* = 0.0001 for mi-NC vs. *miR-200a-3p*) and invasive (Fig. 3e, *P* = 0.0013 for mi-NC vs. *miR-141-3p*, *P* = 0.0072 for mi-NC vs. *miR-200a-3p*) capabilities of Ishikawa cells were dramatically suppressed in the context of *miR-141-3p* or *miR-200a-3p* overexpression. Finally, the results of western blot analysis demonstrated that EMT progression was inhibited in *miR-141-3p*- or *miR-200a-3p*-overexpressing Ishikawa cells (Fig. 3f). Overall, these results suggested that both *miR-141-3p* and *miR-200a-3p* may exert inhibitory effects during the development of endometriosis.

### YAP1 was a common target of miR-141-3p and miR-200a-3p

**Fig. 4:**
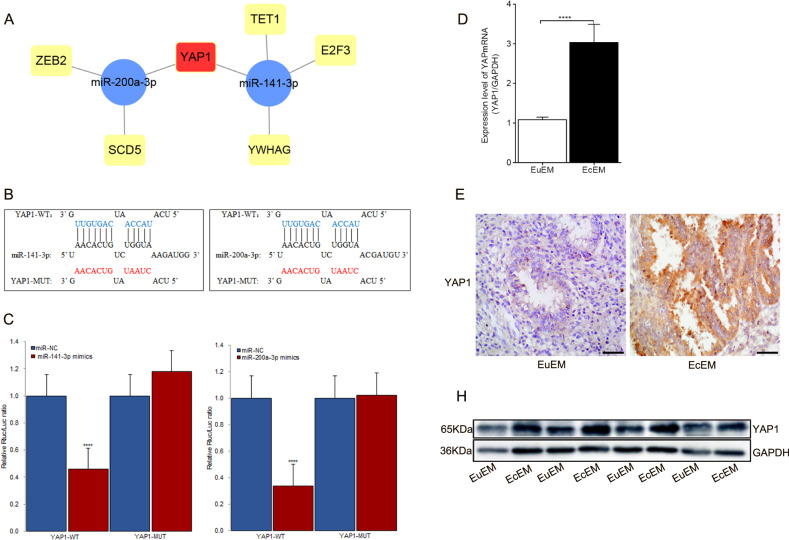
YAP1 is a common target mRNA of miR-141-3p and miR-200a-3p. **a** The downstream target mRNAs of *miR-141-3p/miR-200a-3p* were predicted by three bioinformatics tools (Targetscan, MiRDB, and mirTarbase). **b** The binding sites between 3′UTR of *YAP1* and *miR-141-3p/miR-200a-3p* were predicted by using bioinformatics analysis. **c** Dual-luciferase reporter assays were carried in HEK-293T cells which were co-transfected with *miR-141-3p* mimics or *miR-200a-3p* mimics and pmiR–pmiR-GLO-YAP1. **d** The expression levels of *YAP1* mRNA were tested in ovary endometriosis tissues through using qRT-PCR. **e**, **f** Immunohistochemistry and western blot analysis were used to test YAP1 protein in ovary endometriosis tissues. Immunohistochemistry photographs were taken at ×400 magnification. Scale bars represent 50 μm. ****P < 0.0001 represents statistical difference.

Based on the ceRNA hypothesis, we used MiRWalk v3.0 (http://mirwalk.umm.uni-heidelberg.de/), with strict screening conditions and three prediction algorithms (Targetscan, MiRDB, and mirTarbase), to predict downstream target mRNAs of *miR-141-3p* and *miR-200a-3p*. Four mRNAs were found to be potential targets of *miR-141-3p*, whereas three mRNAs were found to be potential targets of *miR-200a-3p* (Fig. 4a). Among these seven mRNAs, *YAP1* was the only common target and was chosen for subsequent analyses. The binding sites for *miR-141-3p* and *miR-200a-3p* in the 3′-untranslated region (UTR) of *YAP1* were predicted (Fig. 4b). Similarly, luciferase reporter assays were conducted to demonstrate the interactions between the two miRNAs and *YAP1*. The luciferase activity of wild-type *YAP1* (YAP1-WT) was significantly reduced in HEK-293T cells transfected with *miR-141-3p* mimics (*P* = 0.00001) or *miR-200a-3p* mimics (*P* = 0.00001), whereas that of mutated *YAP1* (YAP1-MUT) was not significantly altered (Fig. 4c). Subsequently, high expression levels of YAP1 mRNA (*P* = 0.00004) and protein (*P* = 0.013) were detected in EcEM tissues compared with that in EuEM tissues (Fig. 4d–f). Spearman correlation analysis proved negative relevance between YAP1 and *miR-141-3p* (*r* = −0.326, *P* = 0.012) as well as negative relevance between YAP1 and *miR-200a-3p* (*r* = −0.312, *P* = 0.015). On the contrary, *YAP1* expression correlation with *circATRNL1* was proved to be positive (*r* = 0.950, *P* < 0.0001).

### Knockdown of YAP1 inhibited EMT progression in endometriosis

**Fig. 5:**
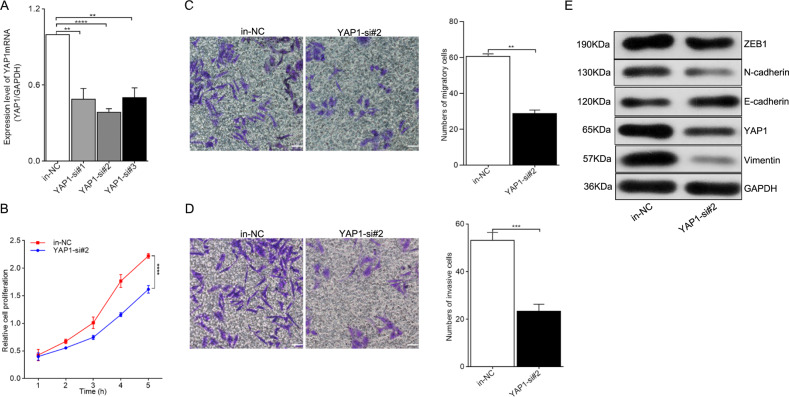
YAP1 silencing inhibited EMT process in endometriosis. **a** Knockdown of *YAP1* expression with *YAP1* siRNA in Ishikawa cells. in-NC was taken as the control group. **b**–**d** CCK-8 and transwell assays were separately carried out to identify the influence of *YAP1 siRNA* on cell proliferation, migration, and invasion. Photographs were taken at ×200 magnification. Scale bar represents 100 μm. **e** The effect of *YAP1 siRNA* on EMT progress in Ishikawa cells was evaluated through detecting the EMT marker proteins with western blot analysis. **P < 0.01, ****P < 0.0001 represent statistical difference.

Three siRNA sequences were constructed and transfected into Ishikawa cells to knockdown *YAP1* mRNA expression; YAP1-si#2 showed the highest inhibitory efficiency as demonstrated through RT-qPCR results (Fig. 5a, *P* = 0.0034 for in-NC vs. YAP1-si#1, *P* = 0.00002 for in-NC vs. YAP1-si#2, and *P* = 0.0026 for in-NC vs. YAP1-si#3) and was selected for further cellular experiments. CCK-8 assays and transwell migration and invasion assays were carried out to evaluate the influence of *YAP1* siRNA on Ishikawa cells. As depicted in Fig. 5b, cell proliferation was obviously inhibited by YAP1-si#2 (*P* < 0.0001). The results of transwell experiments demonstrated that the migratory (Fig. 5c, *P* = 0.0001) and invasive (Fig. 5d, *P* = 0.0013) capabilities of Ishikawa cells were significantly inhibited in the context of *YAP1* knockdown. Finally, the results of western blot analysis demonstrated that EMT progression was inhibited in *YAP1*-silencing Ishikawa cells (Fig. 5e). Overall, these results suggested that *YAP1* may promote EMT progression in endometriosis.

### Regulation of the circATRNL1/miR-141-3p/miR-200a-3p/YAP1 axis in EMT progression in endometriosis

**Fig. 6:**
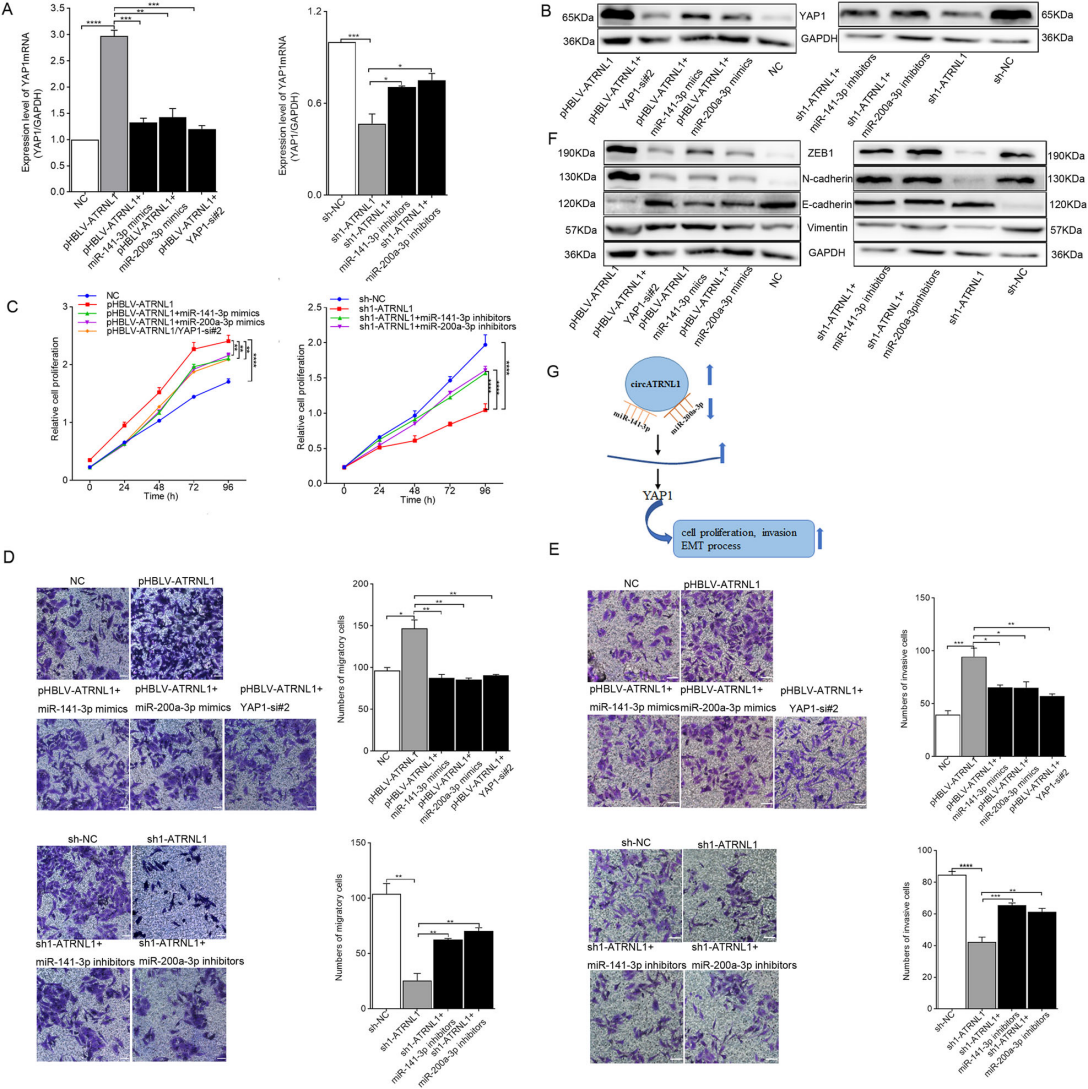
Function of circATRNL1–miR-141-3p/miR-200a-3p–YAP1 axis in endometriosis progression. **a**, **b** mRNA level and protein level of *YAP1* were measured by using qRT-PCR and western blot in Ishikawa cells which were co-transfected with *miR-141-3p/miR-200a-3p* mimics and pHBLV-ATRNL1 or *miR-141-3p/miR-200a-3p* inhibitors and sh-ATRNL1 or *YAP1*-si and pHBLV-ATRNL1. **c**–**e** CCK-8 and transwell assays were separately performed to detect proliferation and motility ability of indicated Ishikawa cells. Photographs were taken at ×200 magnification. Scale bar represents 100 μm. **f** The effect of *circATRNL1–miR-141-3p/miR-200a-3p-YAP1* axis on EMT protein markers in Ishikawa cells was evaluated through western blot analysis. **g** The mechanism diagram was generated to illustrate the mechanism of *circATRNL1–miR-141-3p/miR-200a-3p–YAP1* axis in endometriosis. *P < 0.05, **P < 0.01, ***P < 0.001, ****P < 0.0001 represent statistical difference.

To analyze the regulatory relationships among *circATRNL1*, *miR-141-3p/miR-200a-3p*, and *YAP1*, mRNA and protein levels of *YAP1* were measured, and rescue assays were performed in Ishikawa cells. As depicted in Fig. 6a, b, the increased mRNA and protein levels of *YAP1* caused by pHBLV-ATRNL1 was rescued by *miR-141-3p* or *miR-200a-3p* mimics or *YAP1*-si#2, and meanwhile, *miR-141-3p* or *miR-200a-3p* inhibitors could rescue effects of sh1-ATRNL1 on *YAP1*. According to the results of CCK-8 and transwell assays, the effects of pHBLV-ATRNL1 on promoting proliferation, migration, and invasion in Ishikawa cells could be partly rescued by *miR-141-3p* or *miR-200a-3p* mimics or YAP1-si#2. In addition, *miR-141-3p* or *miR-200a-3p* inhibitors could partly rescue the suppressive effects of silencing *circATRNL1* (Fig. 6c–e). Similarly, western blot results showed that the enhancing effects of pHBLV-ATRNL1 on EMT progression were partly reversed by *miR-141-3p* or *miR-200a-3p* mimics or YAP1-si#2, whereas the inhibitory effects of sh1-ATRNL1 on EMT progression were partly reversed by *miR-141-3p* or *miR-200a-3p* inhibitors (Fig. 6f). Based on these data, we confirmed that *circATRNL1* acted as a ceRNA to modulate endometriosis progression via the *miR-141-3p*/*miR-200a-3p*/*YAP1* axis (Fig. 6g).

## Discussion

In recent years, a growing body of evidence has indicated that circRNAs play crucial regulatory roles in the pathogenesis and development of various diseases, including both malignant and benign diseases^18,19,22–27^. The circRNA *circAF4* functions as an oncogene to regulate the expression of the MLL-AF4 fusion protein and inhibit MLL leukemia progression by sponging *miR-128-3p*^28^. CircRNAs and miRNAs function together to serve as miRNA sponges through a ceRNA network. A recent study revealed that upregulation of *circRNA-100338* was associated with poor prognosis in patients with hepatitis B-related HCC by activating the mammalian target of rapamycin signaling pathway in HCC via the *circRNA-100338/miR-141-3p/RHEB* axis^29^. In a diabetic mouse myocardial fibrosis model, functional experimental results demonstrated that *circRNA-010567* silencing upregulated *miR-141* and downregulated TGF-β1 expression, thus suppressing myocardial fibrosis^30^. *Circ-ZEB1.33* is an oncogene that promotes HCC cell proliferation by enhancing the expression of cyclin-dependent kinases 6 through sponging *miR-200a-3p*^31^. This inspired us to explore the underlying mechanisms and functions of circRNAs in endometriosis.

In this study, we found that compared with the EuEM in patients with endometriosis, *circATRNL1* was significantly elevated in the EcEM. According to the results of IHC and western blot assays, we found decreased E-cadherin and increased ZEB1, N-cadherin as well as vimentin expression in EcEM tissues compared with that in EuEM tissues, which was consistent with previous research results^7,11–13^. To further investigate the influence of *circATRNL1* dysregulation on endometrial epithelial cell activities, a series of functional assays were conducted in Ishikawa cells. The results demonstrated that knockdown of *circATRNL1* inhibited cell proliferation, migration, and invasion, whereas overexpression of *circATRNL1* promoted these cellular bioactivities. Concomitantly, knockdown of *circATRNL1* reduced the protein expression of N-cadherin, vimentin, and ZEB1, but increased the expression of E-cadherin. Therefore, *circATRNL1* may exhibit oncogene-like properties in endometriosis by regulating the EMT process.

Through FISH assays, we first showed that *circATRNL1* was mainly localized in the cytoplasm of Ishikawa cells, which further confirmed our hypothesis that *circATRNL1* may act as a ceRNA by binding with certain miRNAs. Next, bioinformatics analysis and luciferase reporter assays were utilized to identify the target miRNAs of *circATRNL1*. As a result, *miR*-*141*-*3p* and *miR*-*200a*-*3p* were identified. Previous studies have revealed a large number of miRNAs showing expression level alterations between EuEM and EcEM tissues, indicating their involvement in the establishment and progression of endometriosis, such as angiogenesis, inflammation, and immune regulation^32–36^. For example, a recent cell type-specific analysis identified 149 abnormally expressed miRNAs in endometriotic lesions, including extensive upregulation of *miR-139-5p* and downregulation of *miR-375* compared with eutopic stromal cells, which are potentially involved in endometriosis-associated infertility or in the regulation of invasive growth and cell proliferation in endometriosis development^36^. In our study, we detected downregulation of both *miR*-*141*-*3p* and *miR*-*200a*-*3p* in ectopic endometriotic tissues, consistent with previous research^34–36^. Furtherly, a negative expression correlation between circATRNL1 and *miR-141-3p* as well as circATRNL1 and *miR-200a-3p* in endometriosis tissues was separately proved.

Importantly, *miR*-*141*-*3p* and *miR*-*200a*-*3p* belong to the *miR-200* family, which has been reported to regulate the EMT and metastasis by targeting a cohort of target genes, such as ZEB1 and E-cadherin^8,16,37^. Moreover, *miR-200a-3p* and *miR-141-3p* are downregulated in the plasma samples of patients with endometriosis compared with that in healthy controls, suggesting that these miRNAs could be used as putative noninvasive diagnostic biomarkers of endometriosis^38^. Li et al.^39^ found that inhibition of *miR-200a* could promote migration and high mobility group box 1 expression, while decreasing E-cadherin expression in HCC cell lines. Another study showed that EMT progression was inhibited in *miR-200a-3p*-overexpressing papillary thyroid cancer (PTC) cells^40^. Similarly, in this study, we also observed the inhibitory effects of *miR-141-3p* and *miR-200a-3p* on the EMT in endometriosis, and the effects of *circATRNL1* knockdown on cell proliferation, invasion, and EMT suppression could be partially attenuated by *miR-141-3p/miR-200a-3p* inhibition.

MiRNAs regulate gene expression post-transcriptionally by interacting with the 3′-UTRs of mRNAs and triggering either translational repression or mRNA degradation^36^. Through bioinformatics prediction, *YAP1* was found to be the common target of *miR-141-3p* and *miR-200a-3p*. *YAP1* is a downstream oncogene of the Hippo pathway^41^. When activated, *YAP1* localizes to the nucleus and binds to transcription factors, such as TEA domain DNA-binding family of transcription factors (TEAD), and then drives tumor growth, metastasis, and senescence in cancer cell lines and induces the EMT in many types of tumors. *YAP1* induces the EMT in non-small cell lung cancer by regulating the transcription of Slug via interacting with TEAD^42^. In addition, *YAP1* is highly associated with HCC and PTC and frequently upregulated during tumor formation^40,43^. Song et al.^44^ demonstrated that elevated expression of *YAP1* promotes endometrial stromal cell proliferation and blocks apoptosis in vitro and in vivo. In the current study, we found that *YAP1* mRNA and protein levels were positively regulated by *circATRNL1* in endometrial cell lines. The functional assay results furtherly demonstrated that knockdown of *YAP1* expression in Ishikawa cells could significantly inhibited cell proliferation, migration and invasion, and reversed the EMT process. Finally, rescue assays demonstrated that *circATRNL1* improved the EMT by modulating miR-141-3p/*miR-200a-3p*/*YAP1* in endometriosis.

In conclusion, our findings suggested that *circATRNL1* promoted the EMT in endometriosis by upregulating *YAP1* through sponging *miR-141-3p* and *miR-200a-3p*. However, there were some limitations to this study. First, the sample size was relatively small for RT-qPCR, and only rASRM stage III/IV cases were included. Second, the tissue samples were only derived from ectopic cyst walls and EuEM and not from other types of endometriosis or healthy controls. Accordingly, further validation involving in larger cohorts of patients with different types of endometriosis and healthy controls is warranted to confirm the clinical value of *circATRNL1*. Moreover, we used Ishikawa cells rather than primary endometrial epithelial cells for functional experiments, which may lead to unreliable conclusions. Further detailed studies of the mechanisms and functions of *circATRNL1* are needed. With the deepening exploration of circRNAs, we expect to provide new ideas on pathogenesis and find new promising therapeutic targets for endometriosis.

## Supplementary information

Supplementary figure 1

Supplementary figure 1

Supplementary figure 2

Supplementary figure 2
