# Supplementary figures and images for: CircATRNL1 promotes epithelial–mesenchymal transition in endometriosis by upregulating Yes-associated protein 1 in vitro
Source: Cell Death Dis. 2020 Jul 29;11(7):594. doi: 10.1038/s41419-020-02784-4 (PMC7392763; doi:10.1038/s41419-020-02784-4)

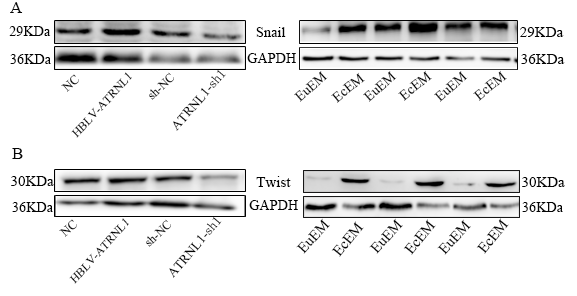

Supplement: Supplementary file 1 — Supplementary figure 1 [file 41419_2020_2784_MOESM1_ESM.tif]

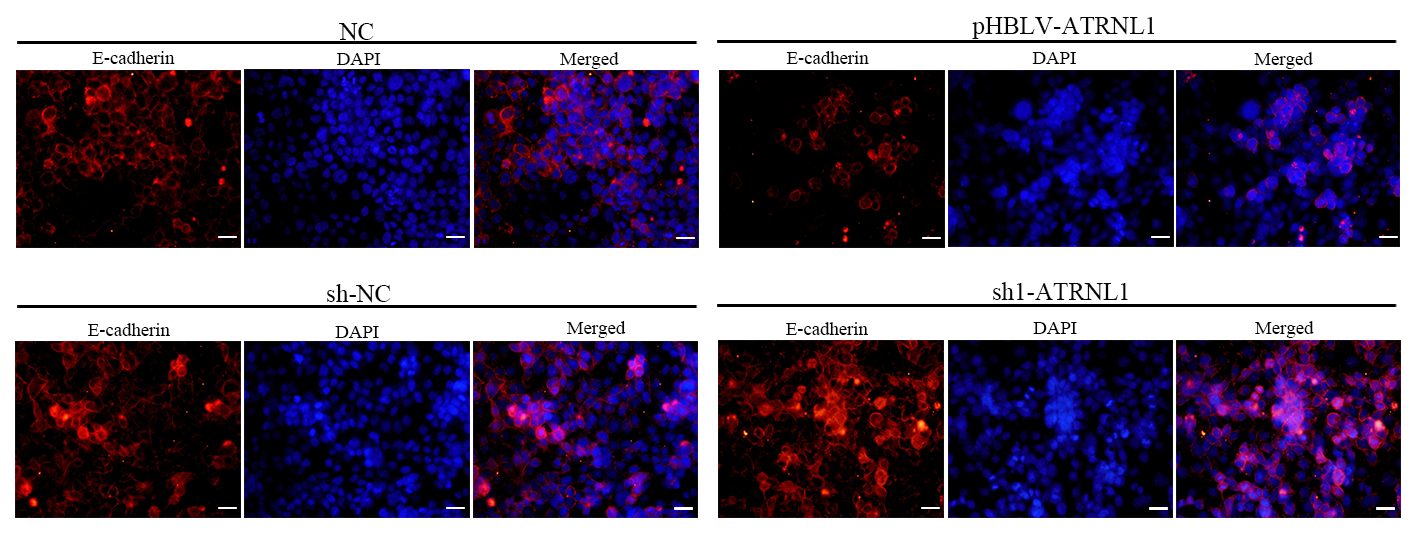

Supplement: Supplementary file 3 — Supplementary figure 2 [file 41419_2020_2784_MOESM3_ESM.tif]
