# Supplementary material for: CircATRNL1 promotes epithelial–mesenchymal transition in endometriosis by upregulating Yes-associated protein 1 in vitro
Source: Cell Death Dis. 2020 Jul 29;11(7):594. doi: 10.1038/s41419-020-02784-4 (PMC7392763; doi:10.1038/s41419-020-02784-4)
Supplement: Supplementary file 2 — Supplementary figure 1 [file 41419_2020_2784_MOESM2_ESM.docx]

**Supplementary Fig. 1**

Expression pattern of Snail and Twist protein in cells and tissues.

(A) Western blot analysis were used to test Snail protein in ovary endometriosis tissues in Ishikawa cells transfected with *circATRNL1* lentivirus vectors.

(B) Western blot analysis were used to test Twist protein in ovary endometriosis tissues in Ishikawa cells transfected with *circATRNL1* lentivirus vectors.
