# Supplementary material for: CircATRNL1 promotes epithelial–mesenchymal transition in endometriosis by upregulating Yes-associated protein 1 in vitro
Source: Cell Death Dis. 2020 Jul 29;11(7):594. doi: 10.1038/s41419-020-02784-4 (PMC7392763; doi:10.1038/s41419-020-02784-4)
Supplement: Supplementary file 4 — Supplementary figure 2 [file 41419_2020_2784_MOESM4_ESM.docx]

**Supplementary Fig. 2**

Immunofluorescence was utilized to analyze the impacts of *circATRNL1* overexpression or knockdown on EMT progress in Ishikawa cells. Photographs were taken at 400× magnification. Scale bars represent 20 μm.
